# Tasting arterial blood: what do the carotid chemoreceptors sense?

**DOI:** 10.3389/fphys.2014.00524

**Published:** 2015-01-14

**Authors:** Nanduri R. Prabhakhar, Michael J. Joyner

**Affiliations:** ^1^Institute for Integrative Physiology, Center for Systems Biology of O_2_ Sensing, The University of ChicagoChicago, IL, USA; ^2^Department of Anesthesiology, Mayo ClinicRochester, MN, USA

**Keywords:** glomus cells, K^+^ channels, carbon monoxide, hydrogen sulfide, hypoglycemia, diabetes

## Abstract

The carotid bodies are sensory organs that detect the chemical composition of the arterial blood. The carotid body sensory activity increases in response to arterial hypoxemia and the ensuing chemoreflex regulates vital homeostatic functions. Recent studies suggest that the carotid bodies might also sense arterial blood glucose and circulating insulin levels. This review focuses on how the carotid bodies sense O_2_, glucose, and insulin and some potential implications of these sensory functions on physiological regulation and in pathophysiological conditions. Emerging evidence suggests that carbon monoxide (CO)-regulated hydrogen sulfide (H_2_S), stemming from hypoxia, depolarizes type I cells by inhibiting certain K^+^ channels, facilitates voltage-gated Ca^2+^ influx leading to sensory excitation of the carotid body. Elevated CO and decreased H_2_S renders the carotid bodies insensitive to hypoxia resulting in attenuated ventilatory adaptations to high altitude hypoxia, whereas reduced CO and high H_2_S result in hypersensitivity of the carotid bodies to hypoxia and hypertension. Acute hypoglycemia augments the carotid body responses to hypoxia but that a prolonged lack of glucose in the carotid bodies can lead to a failure to sense hypoxia. Emerging evidence also indicates that carotid bodies might sense insulin directly independent of its effect on glucose, linking the carotid bodies to the pathophysiological consequences of the metabolic syndrome. How glucose and insulin interact with the CO-H_2_S signaling is an area of ongoing study.

## Introduction

Based on the anatomical location and morphology, Frenando De Castro proposed that the function of the carotid body is to detect “…..chemical composition of the blood (chemical sensing) and the information is transmitted to the nerve terminals which by reflex action will influence on the functional activity of other organs” (De Castro, 1926). Independent studies by Jean-Francois Heymans and Corneille F. Heymans established that the carotid sinus region is divided into two different portions, the carotid body (glomus) which is stimulated by the chemical composition of the arterial blood, whereas the carotid sinus is the seat of the pressor receptors (Heymans and Heymans, 1927). Subsequently several investigators examined the effects of hypoxemia (i.e., reduced O_2_ levels in arterial blood) on the carotid body. It is now established that hypoxemia increases carotid body sensory nerve activity and the ensuing reflex regulates cardio-respiratory functions (see Fidone and Gonzalez, 1986; Fitzgerald and Lahiri, 1986; Gonzalez et al., 1994; Kumar and Prabhakar, 2012 for references). Recent studies indicate that carotid bodies might also sense changes in arterial blood glucose and insulin levels (Koyama et al., 2000; Wehrwein et al., 2010; Ribeiro et al., 2013; Limberg et al., 2014). This brief review will focus on how the carotid bodies sense changes in arterial blood O_2_, and emerging information about their role as sensors of glucose and insulin. The implications of these sensory functions of the carotid body on physiological regulation and in pathophysiological conditions will be highlighted.

## Anatomical location and morphology of the carotid body

The carotid bodies are situated bilaterally at the bifurcation of the common carotid artery. The anatomical location of the carotid bodies favors detecting the changes in the arterial blood composition before the stimulus reaches the brain which is highly depdendent on oxygen and glucose for sustained function. Sensory innervation to the carotid body is provided by a branch of the glossopharengeal nerve called the “carotid sinus nerve (CSN).” The cell bodies of the CSN reside in the petrosal ganglion. Autonomic innervation comes from the post-ganglionic fibers of the superior cervical ganglion. The carotid body receives the highest blood flow per tissue weight of any organ in the body, with a value of approximately 1000–2000 ml min^−1^ 100 g^−1^ (De Burgh Daly et al., 1954; Clarke et al., 1986; Barnett et al., 1988), which is two- to four-fold higher than the blood flow to the heart during heavy exercise (Duncker and Bache, 2008). The chemoreceptor tissue is composed of two major cell types: the type I (also called glomus) cells and type II cells. A substantial body of evidence suggests that type I cells are the initial sites of sensory transduction and they work in concert with the nearby afferent nerve ending as a “sensory unit;” whereas the type II cells are supporting cells resembling glial cells of the nervous system (see Kumar and Prabhakar, 2012 for references).

## Characteristics of the carotid body sensory nerve response to hypoxia

The sensory discharge of the carotid sinus nerve is low under normoxia (arterial PO_2_ ~100 mmHg), which increases dramatically even with a modest drop in arterial PO_2_ (e.g., 80–60 mmHg; Eyzaguirre and Lewin, 1961; Hornbein et al., 1961; Biscoe et al., 1970; Vidruk et al., 2001). The response is fast and occurs within seconds after the onset of hypoxia (Black et al., 1971; Ponte and Purves, 1974). Because of its high blood flow and exquisite sensitivity to hypoxia, the carotid body is uniquely suited to sense and respond to even a modest drop in PO_2_.

## Role of gaseous messengers in the carotid body response to hypoxia

Emerging evidence suggests that the gaseous messengers carbon monoxide (CO) and hydrogen sulfide (H_2_S) play a critical role in hypoxic sensing by the carotid body (Prabhakar, 2013). The following section describes how CO and H_2_S contribute to the O_2_ sensing by the carotid body.

### Carbon monoxide (CO)

Glomus cells express heme oxygenase-2 (HO-2), an enzyme that catalyzes the formation of CO (Prabhakar et al., 1995), and CO is a physiological inhibitor of the carotid body and glomus cell response to hypoxia (Prabhakar et al., 1995; Williams et al., 2004; Peng et al., 2014). CO levels are high during normoxia, and hypoxia decreases CO levels in a stimulus-dependent manner in the carotid body (Peng et al., 2014). These findings demonstrate that changes in O_2_ levels are transduced to changes in CO production in the chemoreceptor tissue.

### Hydrogen sulfide (H_2_S)

It is being increasingly recognized that H_2_S is another gaseous messenger that participates in physiological functions (Yang et al., 2008). Whilst O_2_ levels regulate CO, CO itself does not trigger the sensory excitation of the carotid body. Instead, CO contributes to the carotid body sensory excitation by regulating H_2_S production from the enzyme, cystathionine-γ-lyase (CSE, Peng et al., 2010, 2014). CO suppresses H_2_S levels by inhibiting CSE (Peng et al., 2010, 2014). As a consequence, during normoxia high CO levels are associated with low H_2_S levels; whereas during hypoxia, low CO levels are accompanied with high H_2_S levels, paralleling the sensory nerve excitation (Peng et al., 2010, 2014). Either genetic deletion or pharmacological blockade of CSE result in marked suppression of H_2_S generation during hypoxia (Peng et al., 2010) leading to remarkable blunting of hypoxia-evoked sensory nerve excitation and ventilatory stimulation (Peng et al., 2010). These findings suggest that H_2_S mediates the carotid body sensory nerve excitation by hypoxia.

How might H_2_S contribute to carotid body excitation by hypoxia? The general consensus is that hypoxia depolarizes glomus cells by inhibiting certain K^+^ channels leading to Ca^2+^-dependent release of excitatory neurotransmitter(s), which stimulates the afferent nerve ending and increases the sensory nerve activity (Gonzalez et al., 1994; Kumar and Prabhakar, 2012; Moya et al., 2012; Nurse and Piskuric, 2012; Prabhakar and Peers, 2014). The following lines of evidence suggest that H_2_S mediates hypoxia-induced glomus cell depolarization and voltage-gated Ca^2+^ influx: (a) like hypoxia, H_2_S donor (NaHS) inhibits maxi-K^+^ (Li et al., 2010; Telezhkin et al., 2010), TASK like K^+^ channel activities and depolarizes type I cells (Buckler, 2012), (b) hypoxia-evoked Ca^2+^ influx is markedly reduced or absent in CSE null glomus cells or after pharmacological blockade of H_2_S synthesis (Makarenko et al., 2012), (c) H_2_S donor (NaHS) elevates [Ca^2+^]_*i*_ in glomus cells and this effect was absent in the absence of extracellular Ca^2+^ (Buckler, 2012; Makarenko et al., 2012) as well as by preventing the depolarization by voltage-clamping the cell at the resting membrane potential (Buckler, 2012), and (d) nifedipine, a blocker of L-type Ca^2+^ channel, prevents H_2_S-as well as hypoxia-evoked [Ca^2+^]_*i*_ elevation in glomus cells (Makarenko et al., 2012). In addition, H_2_S donor increases NADH auto fluorescence in glomus cells suggesting that H_2_S might mediate its actions in part due to its effects on the mitochondrial electron transport chain (Buckler, 2012). These studies taken together suggest that CO-regulated H_2_S, stemming from hypoxia, depolarizes type I cells by inhibiting certain K^+^ channels, facilitates voltage-gated Ca^2+^ influx and thus produces sensory excitation of the carotid body.

## Impact of inherent variations in CO-H_2_S signaling on the carotid body O_2_ sensing

The chemosensory reflex is a critical regulator of breathing, sympathetic tone, and blood pressure (Fitzgerald and Lahiri, 1986; Kumar and Prabhakar, 2012). However, healthy human subjects exhibit substantial variations (about three-fold) in the chemosensory reflex as evidenced by variations in the ventilatory response to hypoxia (Weil, 2003). Such variations were also reported in rodents. For instance, in comparison to Sprague-Dawley (SD) rats, Brown-Norway (BN) rats display a markedly reduced ventilatory response to hypoxia (Strohl et al., 1997; Hodges et al., 2002), while Spontaneous Hypertensive (SH) rats exhibit an augmented one (Hayward et al., 2012). A recent study examined whether variations in the chemosensory reflex are due to differences in O_2_ sensing by the carotid body in BN, SH, and SD rats (Peng et al., 2014). BN carotid bodies exhibited severely impaired glomus cell and sensory nerve responses to hypoxia, whereas SH rat carotid bodies showed augmented hypoxic response as compared with SD rats.

The low hypoxic sensitivity in the BN carotid body was associated with high CO and low H_2_S levels; whereas, the augmented hypoxic sensitivity of SH rat carotid body was accompanied with low CO and high H_2_S levels under both normoxia and hypoxia, respectively as compared with SD carotid bodies. The altered CO and H_2_S levels in BN and SH rats was not associated with the changes in HO-2 and CSE proteins in glomus cells (Peng et al., 2014). Remarkably, treating BN carotid bodies with a heme oxygenase inhibitor decreased CO levels, increased basal and hypoxia-induced H_2_S levels, and restored the magnitude of the hypoxic sensitivity, which was comparable to SD rats. Treating SH rat carotid bodies with a CO donor or a CSE inhibitor reduced H_2_S levels and attenuated the hypoxic sensitivity (Peng et al., 2014). These findings suggest that high CO and low H_2_S contribute to inherent hyposensitivity of the carotid body to hypoxia; whereas, low CO and high H_2_S leads to hypersensitivity of the carotid body to hypoxia, further supporting CO-regulated H_2_S governs hypoxic sensing by the carotid body.

## Physiological implications of carotid body O_2_ sensing

### Consequences of hyposensitivity of the carotid body to hypoxia

BN rats exhibited reduced hypoxic ventilatory response (HVR) and near absence of hypoxia-evoked sympathetic nerve activity compared to SD rats (Peng et al., 2014). High-altitude hypoxia leads to a carotid body-mediated increase in breathing, or ventilatory adaptation to hypoxia (VAH) (Dempsey and Forster, 1982). A diminished HVR can result in attenuated VAH (Dempsey and Forster, 1982) and high-altitude pulmonary edema (Hackett et al., 1988; Matsuzawa et al., 1989; Hohenhaus et al., 1995). BN rats exposed to hypobaric hypoxia simulating 8500 m altitude for 16 h showed remarkable absence of VAH and profound pulmonary edema (Peng et al., 2014). Treating BN rats with a heme oxygenase inhibitor, improved ventilatory and sympathetic nerve responses to hypoxia, restored VAH and prevented hypobaric hypoxia-induced pulmonary edema (Peng et al., 2014).

### Consequences of hypersensitivity of the carotid body to hypoxia

**Figure 1:**
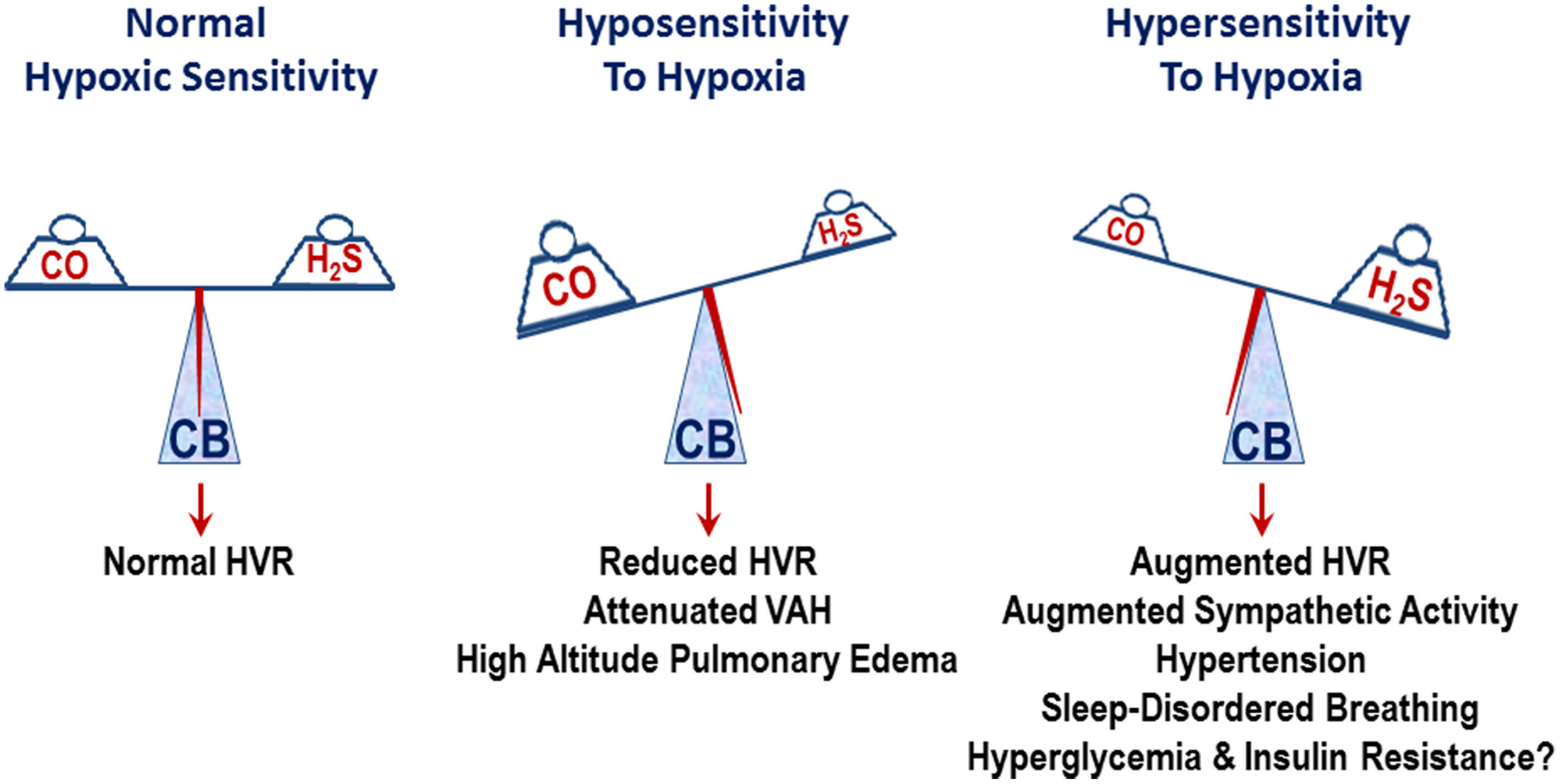
**Schematic presentation of the impact of inherent variations in carbon monoxide (CO) and hydrogen sulfide (H_2_S) levels on the carotid body (CB) sensitivity to hypoxia and its influence on physiological responses**. HVR, hypoxic ventilatory response; attenuated VAH, ventilatory adaptation to hypoxia.

Spontaneous hypertensive (SH) rats (Przybylski, 1978) and human subjects with essential hypertension exhibit augmented ventilatory responses to hypoxia (Trezebski et al., 1983) and these effects were attributed to enhanced carotid body sensitivity to low O_2_ (Przybylski, 1981; Trezebski et al., 1983). Later studies showed that the carotid body response to hypoxia is indeed augmented in SH rats (Fukuda et al., 1987) and carotid body chemoreflex mediates the heightened sympathetic nerve activity in SH rats (Tan et al., 2010). Based on these findings, Paton and co-workers (Abdala et al., 2012) tested whether ablation of the carotid bodies normalizes blood pressures in SH rats. They found that chronic bilateral sectioning of the carotid sinus nerves substantially lowered blood pressures in SH rats. Since L-propargylglycine (L-PAG), an inhibitor of H_2_S synthesis, reduced the hypersensitivity of the carotid body in SH rats, Peng et al. (2014) examined whether L-PAG treatment affect the age-dependent development of hypertension in SH rats. Five-week-old SH rats were treated with either vehicle (saline) or L-PAG every day, with blood pressures measured every week for 5 weeks. Compared to vehicle-treated SH rats, L-PAG-treated SH rats presented a pronounced reduction in blood pressures. Ablation of the carotid bodies from 5-week-old SH rats also attenuated age-dependent hypertension to the same extent as L-PAG treatment. However, treating carotid body ablated rats with L-PAG caused no further decline in blood pressures, suggesting that the carotid bodies are the likely sites of action of L-PAG. These findings underscore a mechanism through which elevated H_2_S signaling in the carotid body contributes to the hypoxic hypersensitivity and progression of hypertension in SH rats. The impact of inherent variations in CO-H_2_S signaling on carotid body O_2_ sensing and their consequences on physiological responses is schematically illustrated in Figure 1.

## The carotid bodies: expanding role in human physiology and pathophysiology?

In the above sections, we briefly reviewed the sensory transduction mechanisms that link carotid body function with hypoxia and also showed that stimulation of the carotid bodies by hypoxia is a key driver of ventilation and sympathetic nerve activity. Additionally, the data from the congestive heart failure (CHF) rats provide evidence that tonic or perhaps “overactive” stimulation of the carotid bodies in the absence of hypoxia might be an important driver of elevated sympathetic activity in a number of circumstances (Del Rio et al., 2013). In this context, there is evidence from animal models and also human studies suggesting that the carotid bodies are tonically active during normoxia and drive the increased sympathetic nerve activity in some patients with heart failure and also chronic kidney disease (Paton et al., 2013). In fact, excessive ventilation during exercise in heart failure patients is likely driven in part by hypersensitive carotid bodies and is associated with poor patient outcomes (Ponikowski et al., 2001). Hyperoxia or dopamine infusions to “turn off” the carotid body function in humans and in dogs can also blunt the sympathoexcitatory responses to a number of stressors like stimulation of metabosensitive skeletal muscle afferents during exercise (Ponikowski et al., 2001). Together these and other observations suggest that the role of the carotid body extends beyond O_2_ sensing. These findings also suggest that carotid body sensory activity might be elevated in the normoxic state during hypertension, CHF and/or insulin resistance. A key questsion is whether this tonic activation is due to the enhanced CO-H_2_S signaling in the carotid body under these conditions or some other mechanism(s).

## What about glucose and hypoxia?

Over the last fifteen or so years evidence from a variety of models has suggested that the carotid bodies either directly sense arterial blood glucose concentrations or that prevailing glucose levels can influence the stimulus response curve of the carotid body to hypoxia. Pardal and López-Barneo (2002) reported that hypoglycemia stimulates glomus cells and enhances their responses to hypoxia. A recent study showed that glycogen depletion of the carotid body can lead to a brief period of hyperresponsiveness followed by hyporesponsiveness to hypoxia (Holmes et al., 2014). These findings are mirrored earlier observations in humans showing that maneuvers that cause whole body glycogen depletion initially stimulate ventilation, but that days of “semi-starvation” blunt the ventilatory responses to hypoxia but not to hypercapnia (Heigenhauser et al., 1983; Lindholm and Gennser, 2005). These studies indicate that acute hypoglycemia augments the carotid body responses to hypoxia, but a prolonged lack of glucose in the carotid bodies can lead to a failure to sense hypoxia.

## What about glucose *per se*?

The data presented above also raises questions whether carotid body can be sensors of blood glucose levels *per se*. As discussed earlier, this makes some teleological sense given the dependence of the brain on blood glucose and the location of the carotid bodies as sentinels in the arterial circulation just proximal to the brain. When the question is posed this way a number of interesting observations from *in vivo* studies are available. First, the counter regulatory hormonal responses to hypoglycemia induced by the insulin clamp technique in dogs are blunted in animals that have undergone carotid body resection (Koyama et al., 2000). Likewise, hyperoxia in humans (again to acutely “turn off” the carotid bodies) can also blunt the counter regulatory responses normally seen during hyperinsulinemic, hypoglycemic clamps (Wehrwein et al., 2010). There is also observational evidence in patients with COPD that correcting their arterial hypoxemia alters whole body glucose homeostasis in a manner consistent with the idea that the carotid body plays a role in the regulation of blood glucose (Jakobsson and Jorfeldt, 2006). Similar conclusions can also be drawn in diabetic patients on insulin who have received hyperbaric therapy for wound healing. In these patients, the risk of hypoglycemia seems increased during the hyperbaric treatment suggesting that it interferes with the ability of the carotid bodies to sense blood glucose (Al-Waili et al., 2006).

While the observations cited above are consistent with the idea that the carotid bodies also sense glucose, it should however, be noted that not all studies provide strong support for hypoglycemia as a stimulus for the carotid boides (Bin-Jaliah et al., 2004; Conde et al., 2007; Gallego-Martin et al., 2012). This could be due to technical or experimental design issues or complex interactions between glucose and insulin that we will discuss when we consider insuin as a potential stimulator of the carotid bodies.

How any sensing of blood glucose at the cellular level might differ or intersect with the hypoxic sensing via the CO-H_2_S signaling or other mechanisms is currently unclear. Additionally, how carotid body regulation of blood glucose and sympathetic activty might be amplified in conditions like obstructive sleep apnea which is associated with both hypertension and diabetes are also unclear. This is especially important because tonically high levels of endogenous glucose production are a hallmark of type 2 diabetes and it seems reasonable to hypothesize that this might be driven in part by hyperresponsive carotid body (Basu et al., 2004).

## A role for insulin?

In a number of the *in vivo* and human studies mentioned above, hypoglycemia was generated using insulin infusions. Addtionally, in patients with type 2 diabetes insulin levels are generally higher for longer periods of time than in healthy subjects. In this context, insulin also has powerful sympathoexcitatory properties, and there is some evidence that it can stimulate ventilation (Ward et al., 2007). Furthermore, recent studies in rodents have identified insulin receptors on the glomus cells and linked the carotid body to a variety of the pathophysiological consequences of the metabolic syndrome (Ribeiro et al., 2013; Limberg et al., 2014). Together these observations suggest that insulin might stimulate the carotid body independently of changes in glucose. In fact, we have recently argued that periodic fluctuations in insulin in the context of the metabolic syndrome might be the “new” intermittent hypoxia (Limberg et al., 2014).

## Summary and future directions

Great progress has been made in the last two decades on how glomus cells in the carotid body sense and transduce arterial oxygen levels. Understanding of the broad based phsyiological and pathophysiological repsonses evoked by the carotid body stimulation has also grown dramatically. All this new information has led to exciting new opportunities to investigate how insulin and glucose interact both acutely and chronically with the carotid body during both normoxia and hypoxia including intermittent hypoxia. This area is also ripe for translational research and team science linking the cellular mechanisms and adaptations, interogated *in vitro* with *in vivo* models including studies in humans. At a fundatmental level, understanding how various conditions associated with carotid body stimulation interact with the CO-H_2_S pathway in the sensing of hypoxia will be of great interest. From a translational perspective it seems reasonable at this time to ask how much of the pathophysiology of sleep apnea/metabolic syndrome diad is being driven or amplified by the carotid body?

### Conflict of interest statement

The authors declare that the research was conducted in the absence of any commercial or financial relationships that could be construed as a potential conflict of interest.
